# With Friends Like These: The Complex Role of Neutrophils in the Progression of Severe Pneumonia

**DOI:** 10.3389/fcimb.2017.00160

**Published:** 2017-05-01

**Authors:** Roger D. Pechous

**Affiliations:** Department of Microbiology and Immunology, University of Arkansas for Medical SciencesLittle Rock, AR, USA

**Keywords:** pneumonia, neutrophils, pulmonary damage, pulmonary immunity, pulmonary infection

## Abstract

Pneumonia is a leading cause of death from infection in the United States and across the globe. During pulmonary infection, clear resolution of host inflammatory responses occurs in the absence of appreciable lung damage. Neutrophils are the first wave of leukocytes to arrive in the lung upon infection. After activation, neutrophils traffic from the vasculature via transendothelial migration through the lung interstitium and into the alveolar space. Successful pulmonary immunity requires neutrophil-mediated killing of invading pathogens by phagocytosis and release of a myriad of antimicrobial molecules, followed by resolution of inflammation, neutrophil apoptosis, and clearing of dead or dying neutrophils by macrophages. In addition to their antimicrobial role, it is becoming clear that neutrophils are also important modulators of innate and adaptive immune responses, primarily through the release of cytokines and recruitment of additional waves of neutrophils into the airways. Though typically essential to combating severe pneumonia, neutrophil influx into the airways is a double-edged sword: Overzealous neutrophil activation can cause severe tissue damage as a result of the release of toxic agents including proteases, cationic polypeptides, cytokines, and reactive oxygen species (ROS) aimed at killing invading microbes. In extreme cases, the damage caused by neutrophils and other innate immune mediators become the primary source of morbidity and mortality. Here, we review the complex role of neutrophils during severe pneumonia by highlighting specific molecules and processes that contribute to pulmonary immunity, but can also drive progression of severe disease. Depending on the identity of the infectious agent, enhancing or suppressing neutrophil-mediated responses may be key to effectively treating severe and typically lethal pneumonia.

## Introduction

Pulmonary infection with bacterial, viral, or fungal pathogens can lead to the massive infiltration of innate immune populations, primarily polymorphonuclear leukocytes (PMNs), into the airways and alveolar spaces. Defined as pneumonia, this inflammatory response is aimed at controlling and clearing invading pathogens, but can severely compromise pulmonary function, resulting in significant mortality. Pneumonia lags only heart disease as the greatest burden of disease worldwide, and is the leading cause of death from infection in the United States (Mizgerd, 2006, 2008). Pulmonary infection is the most common reason for American children to be hospitalized, and for Americans 65 years and older roughly half of infectious disease-related hospitalizations and deaths are from pneumonia (Mizgerd, 2006). Particularly in its later stages, severe pneumonia can be very difficult to treat clinically, even in otherwise healthy patients. Despite administration of appropriate therapy, mortality rates can still approach 50% in patients with severe pneumonia (Restrepo and Anzueto, 2009). This is primarily due to the dysregulation of the very host responses aimed at eliminating invading pathogens. Though typically essential to combating infection, the inability to appropriately control these responses results in toxic host inflammation that complicates treatment (Mizgerd, 2008; Bordon et al., 2013). Thus, the outcome of severe pneumonia depends on the appropriate balance between effective microbial clearance and the magnitude of host inflammatory responses (Mizgerd, 2008; Rendon et al., 2016). A lack of understanding of the factors underlying the dysregulation of host innate immune responses is a significant barrier to enhancing survivability of severe pneumonia.

Key to the progression of pneumonia are neutrophils, the cell type most associated with the development of severe disease. Neutrophils migrate directly to the site of infection, where they accumulate in significant numbers and unleash a torrent of antimicrobial factors aimed at controlling and clearing infection. Though neutrophils are essential mediators of pulmonary immunity, they can also contribute to the development of severe disease. The very molecules and processes that make neutrophils such potent innate immune responders can cause a great deal of host tissue damage, and can contribute significantly to disease pathology and severity. In this review, we discuss the complex role neutrophils play in the pathogenesis of severe pneumonia, examining their function as mediators of both pulmonary immunity and pulmonary damage. We highlight key molecules and processes employed by neutrophils to destroy invading pathogens, and the unintended consequences that can result from their dysregulation. The processes that govern neutrophil-mediated immunity are wide-ranging and complex, each with an extensive literature beyond the scope of this article. We touch briefly on a handful of well-established factors shown to contribute to both clearance of invading pathogens and the onset of pulmonary damage that defines the pathogenesis of disease. What emerges is an intriguing and complex story, where a key innate immune population is essential to combating infection, but can also be responsible for pathogenesis and mortality of the very disease it is tasked with combating.

## Pulmonary infection, immunity, and neutrophils

**Figure 1:**
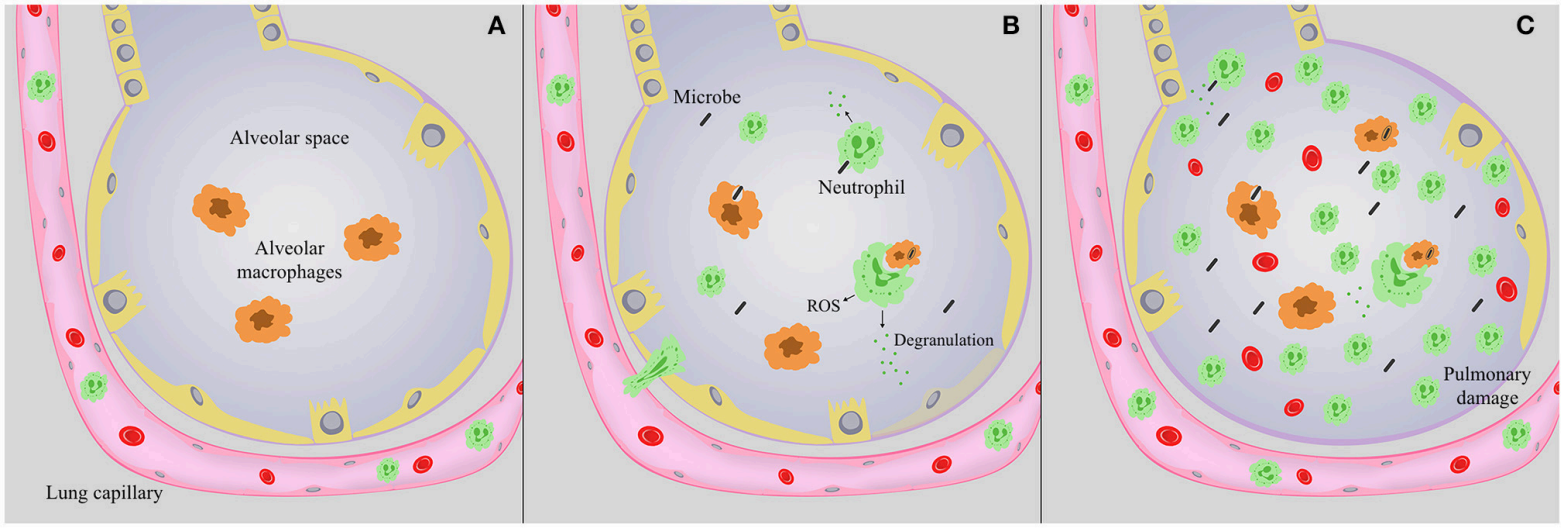
**Lung alveoli during infection. (A)** Under steady state conditions, alveolar macrophages (orange) survey the alveolar space, and neutrophils are present in the pulmonary vasculature. **(B)** During infection, neutrophils (green) migrate into the alveolar space, where they can degranulate, facilitate an oxidative burst, and along with alveolar macrophages phagocytose and kill invading microbes. Antimicrobial peptides and enzymes are released during degranulation, but cause minimal tissue destruction that is ultimately repaired. **(C)** During severe pneumonia, as invading microbes persist neutrophils continue to flood the airways, potentially releasing a myriad of antimicrobial peptides, enzymes and ROS. As infection progresses this process continues to the detriment of the host, causing excessive tissue damage and disrupting the epithelial-capillary barrier, resulting in hemorrhage, edema, and compromise of pulmonary function.

Infection with a wide range of microbes can cause pneumonia. Upon inhalation, invading pathogens encounter a variety of antimicrobial roadblocks, including the mucociliary escalator, surfactant protein, and lung antimicrobial peptides. Upon deposition of a microbe in the alveoli, alveolar macrophages are the sentinel cell type tasked with identifying a potential threat. These cells survey the alveolar space, phagocytose and kill invading microbes, and initiate downstream immune signaling. Though key to combating infection, neutrophils are not a major component of the airway compartment, but are present patrolling the pulmonary vasculature during steady state conditions (Figure 1; Kreisel et al., 2010; Kolaczkowska and Kubes, 2013). Alveolar macrophages, along with pulmonary epithelial and endothelial cells, can induce a wave of cytokines and chemokines in order to sequester circulating neutrophils in the lung, activate resting neutrophils, and stimulate neutrophil proliferation in the bone marrow (Nathan, 2006; Craig et al., 2009; Kolaczkowska and Kubes, 2013). Alveolar macrophage secretion of chemokines such as IL-8 and CXCL5 are responsible for a great deal of neutrophil chemotaxis into the lung (Osman et al., 1998; Jeyaseelan et al., 2004; Mei et al., 2010; Grommes and Soehnlein, 2011). In addition, pulmonary endothelial and epithelial cells release a myriad of signaling molecules in response to infection that activate and recruit neutrophils to the alveolar space. These include cytokines and chemokines including IL-8 and CXCL5, and lipid metabolites such as the eicosanoid hepoxilin A_3_ (Smart and Casale, 1994a,b; Jeyaseelan et al., 2005; Bhowmick et al., 2013). These signals also dramatically increase numbers of circulating neutrophils, enlarging the available pool for extravasion into pulmonary tissue (Craig et al., 2009). After activation, circulating neutrophils traffic from pulmonary capillaries via transendothelial migration, through the interstitium, and into the alveolar space (Figures 1B,C). During migration, neutrophils are primed or activated in preparation of encountering invading microbes. Within pulmonary tissue, neutrophils migrate directly to the site of infection, and armed with an arsenal of antimicrobial tools create a localized inflammatory response aimed at killing potentially pathogenic microbes, and ultimately controlling infection. Successful pulmonary immunity involves macrophage and neutrophil-mediated phagocytosis and killing of invading pathogens; equally important is the subsequent resolution of inflammation. Lung homeostasis is restored through a series of processes that include neutrophil apoptosis and clearance by scavenger macrophages via a process called efferocytosis, followed by repair of any injured bystander tissue. Much of this is mediated through the temporal regulation of specialized pro-resolving mediators such as resolvins that are synthesized from essential fatty acids and facilitate the onset of a resolution phase (Serhan et al., 2002; Chiang et al., 2012). The maintenance of a specific neutrophil lifespan is key to maintaining pulmonary homeostasis. Neutrophils are particularly short-lived cells, lasting roughly 8 h in circulation (Galli et al., 2011; Kolaczkowska and Kubes, 2013). Constitutive neutrophil apoptosis occurs in coordination with their production in the bone marrow to ensure a continuous supply of active cells without overwhelming host tissue (Bordon et al., 2013). Constant turnover of neutrophils by apoptosis is an important aspect to the resolution of inflammation, as it occurs without the release of harmful granule proteins or reactive oxygen species (ROS) (Bordon et al., 2013).

Pathogenic microbes have evolved strategies to evade and/or suppress host inflammatory responses in order to establish infection, acquire nutrients, disseminate to other tissues, and induce spread to additional hosts. The inability to control infection results in the continued accumulation of neutrophils in the airways and the onset of a pro-inflammatory cytokine storm. Thus, as the stimulus persists, neutrophils continue to flood the airways, releasing potent antimicrobial enzymes and reactive oxygen and nitrogen species aimed at killing the microbe that can also damage pulmonary tissue (Figure 1C). In extreme cases the damage caused by neutrophils and other innate immune mediators become the primary source of morbidity and mortality, resulting in severe pneumonia (Nathan and Ding, 2010). The pulmonary damage induced during severe pneumonia ultimately leads to acute respiratory distress syndrome (ARDS) (Narasaraju et al., 2011), a type of acute lung injury (ALI) highlighted by pulmonary edema, alveolar destruction, and eventually total respiratory failure. Key to both resolution of infection and the pathogenesis of ALI are neutrophils.

## The role of neutrophils in combating pulmonary infection

Neutrophils are typically the earliest immune cells recruited to a site of inflammation, and are an essential component of innate immune resistance to respiratory pathogens (Grommes and Soehnlein, 2011; Gomez et al., 2012). Experimentally, depletion of neutrophil populations has been shown to dramatically reduce clearance and exacerbate infection with a number of pathogenic bacteria in the lung, including *Streptococcus pneumoniae, Klebsiella pneumoniae*, and *Legionella pneumophila* (Garvy and Harmsen, 1996; Tateda et al., 2001; Jeyaseelan et al., 2006; Craig et al., 2009). Neutropenia is also known to predispose patients to a myriad of opportunistic lung infections (Dinauer et al., 2000). The effectiveness of neutrophils as mediators of innate immunity is due to their ability to chemotax directly to the site of infection and eliminate invading microbes. Neutrophils are uniquely equipped for this task, harboring a wide range of antimicrobial molecules and processes that have been shown to contribute to host defense during pulmonary infection. Below, we briefly highlight a few well-established factors that are crucial to combating pulmonary infection:

### Neutrophil granules

**Figure 2:**
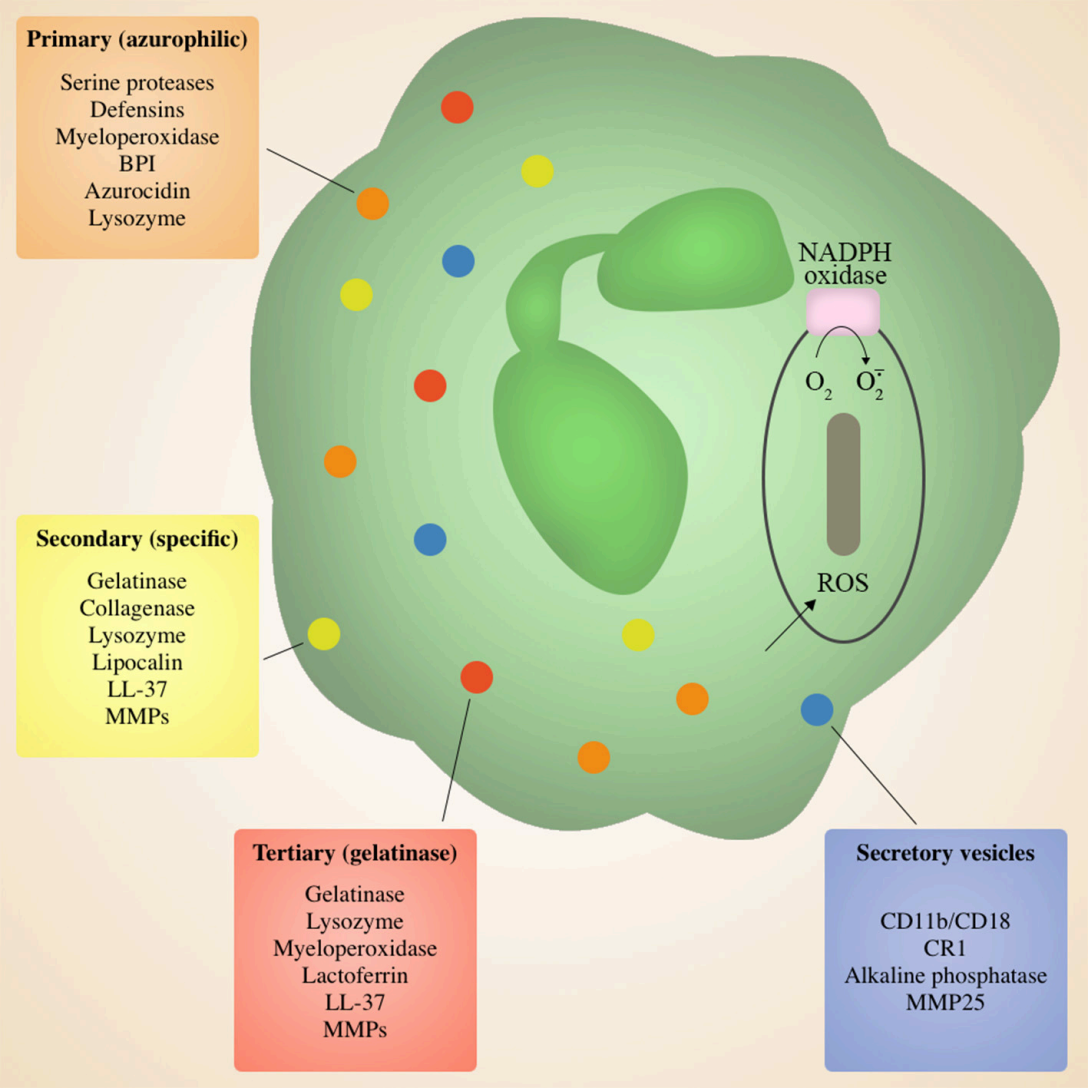
**Neutrophil Granules**. Neutrophils are armed with an arsenal of tools for eliminating invading microbes, including four distinct granules containing a variety of antimicrobial peptides and enzymes, as well as an NADPH oxidase in the phagosomal membrane. Granule contents can be released at the cell surface via degranulation or into the phagolysozome containing a target microbe. Secretory vesicles are the first to be released and primarily contain membrane proteins to facilitate adherence during migration, followed by tertiary granules, and finally primary and secondary granules at the site of inflammation.

During migration and upon deposition in target tissue, neutrophils are primed to release characteristic granules that are involved in the antimicrobial killing of invading pathogens. Neutrophils contain four types of granules differentiated based on protein content: primary (azurophilic), secondary (specific), tertiary (gelatinase) granules, and secretory vesicles (Bordon et al., 2013; Figure 2). Each serve a different purpose, and are released sequentially via exocytosis to the cell surface or to the microbe-containing phagolysosome in response to different signals and/or environmental cues. Secretory vesicles are the first to be released via exocytosis following initial contact of neutrophils with endothelial cells, resulting in the surface expression of key membrane proteins that facilitate rolling through the endothelial monolayer in the blood vessels, thus initiating the process of extravasation to the site of infection (Borregaard et al., 2007; Bordon et al., 2013). This is followed by tertiary granules that are released during migration across the endothelial barrier and into the interstitial space (Grommes and Soehnlein, 2011), and finally primary and secondary granules that are discharged in target tissues (Grommes and Soehnlein, 2011). A majority of granule antimicrobial activity is mediated by release of the contents of primary and secondary granules into the phagolysosome, or degranulation into surrounding tissue. Primary granules contain a myriad of antimicrobial enzymes and peptides including serine proteases and defensins, as well as myeloperoxidase responsible for converting H_2_O_2_ to the antiseptics hypochlorous acid, hypobromous acid and hypoiodous acid (Bainton and Farquhar, 1968a, Bainton and Farquhar, 1968b; Nathan, 2006; Figure 2). Primary granules also release the lipopolysaccharide (LPS)-binding permeability increasing protein (BPI), another potent antimicrobial (Nathan, 2006). Secondary and tertiary granules contain overlapping sets of proteins including lactoferrin, lipocalin, lysozyme, and LL37 (Nathan, 2006), as well as matrix metalloprtoeinases (Bainton and Farquhar, 1968a,b; Nathan, 2006; Figure 2). Effective pulmonary immunity requires the action of the various molecules within granules without inducing significant tissue damage (Mizgerd, 2012). Typically, the neutrophil phagosome containing a putative pathogen fuses with granules to produce a phagolysosome, allowing for direct pathogen exposure to the multiple granule antimicrobial factors. This allows for effective killing of microorganism without the release of potentially damaging granule components into surrounding tissue (Nathan, 2006; Brinkmann and Zychlinsky, 2007).

### Serine proteases

Among the most potent molecules released by primary and secondary granules are neutrophil serine proteases, including elastase, proteinase 3, and cathepsin G (Korkmaz et al., 2010). Neutrophil serine proteases are secreted into the microbe-containing phagolysosome to digest microorganisms in tandem with antimicrobial peptides and ROS (Korkmaz et al., 2010). Neutrophil elastase is a key serine protease that has been demonstrated to contribute to control of invading bacterial and fungal pathogens (Belaaouaj et al., 1998; Tkalcevic et al., 2000; Weinrauch et al., 2002; Hahn et al., 2011). Neutrophil elastase is stored in an active form in primary granules, and is discharged into the phagolysosome following bacterial uptake (Liou and Campbell, 1995). Incubation of elastase with *E. coli* leads to bacterial cell lysis, possibly due to its ability to degrade a number of key outer membrane proteins (Belaaouaj et al., 2000). Elastase has been shown to cleave virulence factors of enterobacteria including *Salmonella enterica, Shigella flexneri*, and *Yersinia enterocolitica*, and likely also does so with pathogens that cause pulmonary infections (Weinrauch et al., 2002). While well-known for combating Gram-negative bacteria, elastase has also been shown to be critical in defense against *S. pneumoniae* infection (Standish and Weiser, 2009; Hahn et al., 2011). Whereas elastase is primarily required for clearance of certain Gram-negative bacteria, cathepsin G has been shown to be effective against infection with Gram-positive bacteria including *Streptococcus pneumoniae* and *Staphylococcus aureus*, as well as certain fungal infections (Tkalcevic et al., 2000; Reeves et al., 2002; Pham, 2006; Standish and Weiser, 2009; Hahn et al., 2011). Proteinase 3 has also been shown to kill bacterial and fungal pathogens through inhibition of protein synthesis and oxygen metabolism (Schreiber et al., 2003; Grommes and Soehnlein, 2011). In addition to direct antimicrobial activity, upon degranulation neutrophil serine proteases can bind key cell surface receptors and modulate chemokine and cytokine activity, thus potentially regulating localized inflammatory processes for optimal antimicrobial activity in the lung (Pham, 2006).

### Antimicrobial peptides

Neutrophil activation also results in the release of a range of cationic polypeptides present within primary, secondary, and tertiary granules that have potent antimicrobial activity. This activity ranges from direct microbial killing to modulation of immune responses to facilitate antimicrobial activity. Lactoferrin stored in secondary and tertiary granules is known to exhibit immune-modulating activity by inducing the production of a range of pro-inflammatory cytokines in nearby cells (Actor et al., 2002). Further, lactoferrin is known to have direct antibacterial, antiviral, and antifungal activity (Levay and Viljoen, 1995). Similarly, the antimicrobial polypeptide LL-37 can activate monocytes and neutrophils directly, and also has broad antimicrobial activity (Aarbiou et al., 2006; Kai-Larsen and Agerberth, 2008; Doss et al., 2010). Defensins are small, cationic peptides stored within primary granules that are known to have antimicrobial properties, primarily by forming pores in the bacterial cell wall (Moraes et al., 2006). The highly positive charge of defensins allows for the disruption of bacterial cell wall integrity in a receptor-independent manner (Moraes et al., 2006). Defensins have also been implicated in modulating inflammation in a receptor-dependent fashion, including by stimulating epithelial cells to produce the neutrophil recruiting cytokine IL-8 through the purinergic receptor P2Y_6_ (Moraes et al., 2006). Their ability to directly kill microbes as well as induce localized inflammatory responses make the neutrophil-derived cationic peptides potent antimicrobials important to multiple stages of pulmonary immunity.

### Reactive oxygen species (ROS)

Important to the initial control of infection, primed neutrophils have a dramatically increased ability to phagocytose microbes and initiate a respiratory burst response at the site of infection. After phagocytosis, neutrophils bombard microbes with ROS within the phagolysozome. Production of ROS including superoxide anion (${\text{O}}_{2}^{-}$) and hydrogen peroxide (H_2_O_2_) is a key component of the innate immune response to infection. The ROS can be potent antimicrobials, likely through the oxidation of proteins, nucleic acids, lipids, and a number of other molecules (Hampton et al., 1998). Neutrophils contain the oxidant-generating enzymes phagocyte NADPH oxidase and myeloperoxidase (Moraes et al., 2006). Phagocyte NADPH oxidase is the main ROS-producing enzyme in neutrophils (Nathan, 2006), and its importance has been realized for some time (Good et al., 1968; Dinauer et al., 2000). Human deficiency of NADPH oxidase is known as chronic granulomatous disease, and results in chronic recurrent infections, and increased susceptibility to lethal infections as a result of the inability to produce ROS (Good et al., 1968; Dinauer et al., 2000) NADPH oxidase pumps large amounts of superoxide into the phagocytic vacuole, where it is converted to H_2_O_2_. Hydrogen peroxide can then be converted by myeloperoxidase to hypochlorite (OCl^−^), which is thought to be primarily responsible for antimicrobial activity, as well as hypochlorous acid (HOCl), hypobromous acid (HOBr), and hydroxyl radical (^.^OH) (Hampton et al., 1998; Brinkmann and Zychlinsky, 2007). Myloperoxidase is not particularly bactericidal on its own, but is known to augment the killing activity of ROS (Klebanoff, 2005; Nathan, 2006). Also, neutrophil NADPH oxidases dramatically alter the pH and ionic compositions of the phagocytic vacuole, and therefore directly influence the killing of microbes by the various enzymes released into the phagolysosomal compartment by granules, particularly the neutrophil serine proteases (Levine and Segal, 2016).

### Nuetrophil extracellular traps (NETs)

In inflammatory conditions neutrophils generate DNA-based neutrophil extracellular traps (NETs) to kill invading microbes (Brinkmann et al., 2004). NET formation is a cell death mechanism by which neutrophils emit DNA “NETs,” large interwoven decondensed chromatin fibers that carry a number of proteins including histones, elastase, MPO, pentraxin, and MMP-9 in an effort to ensnare and eliminate invading microbes. This process, termed “NETosis,” can be induced by a number of inflammatory stimuli including IL-8, LPS, or phorbol myristate acetate (PMA) (Saffarzadeh et al., 2012). Neutrophil elastase and myeloperoxidase also regulate the formation of NETs (Papayannopoulos et al., 2010). The expansive NETs serve as a physical barrier to trap bacteria and prevent dissemination to other tissues. The release of NETs also allows for a highly localized concentration of antimicrobial factors to come into direct contact with trapped pathogens for efficient killing (Brinkmann and Zychlinsky, 2007). The presence of serine proteases within NETs facilitates degradation of key virulence factors to help combat bacterial pathogens (Brinkmann et al., 2004; Papayannopoulos et al., 2010). Deficiencies in both NET release or DNase digestion of NETs have both been shown to increase susceptibility to lethal infection (Beiter et al., 2006; Meng et al., 2012). Gram-positive, Gram negative, and fungal pathogens have all been shown to bind to NETs microscopically (Brinkmann and Zychlinsky, 2007).

### Effector functions

Neutrophils have traditionally been regarded as expert killers but little else, the predominant impression being of bags of destructive enzymes that homed to the site of infection and released their contents shortly before dying. It is now appreciated, though, that neutrophils play a more complex role with regards to their antimicrobial capabilities. Neutrophils have more recently been shown to have the capacity to act as “effector cells,” able to modulate and drive aspects of both innate and adaptive immunity. A number of acquired and innate immune responses in the lungs are shaped by neutrophil-derived signals (Mizgerd, 2008). Neutrophils are known to generate eicosanoids as well as number of key cytokines to influence immune responses and recruit additional neutrophils, including IFN-γ, TNF-a, IL-1, IL-17, and IL-12 (Quinton and Mizgerd, 2015). For example, during pneumococcal infection in mice, IFN-γ secreted by neutrophils is required for immune resistance, and promotes NET formation (Yamada et al., 2011). Neutrophils also generate signals that attract additional antigen presenting cells (APCs), and can influence the differentiation state of macrophages based on what is most beneficial toward controlling a specific infection (Nathan, 2006). Cytokines secreted by neutrophils during infection can also be important for stimulating the trafficking of T cells to infected sites, and therefore the induction of appropriate T cell –mediated responses (Müller et al., 2009). Further, it is becoming clear that neutrophils likely interact with and activate T cells during a number of different conditions, including bacterial infection and chronic inflammatory diseases (Müller et al., 2009). Appropriate neutrophil accumulation and function is also important to induction of necessary adaptive immune responses in the host, highlighting the key and wide ranging role neutrophils play during multiple phases of immune defense in the lung (Nathan, 2006).

## Neutrophils and pulmonary damage: too much of a good thing

Most often, neutrophils are essential components of the innate immune response to pulmonary infection, as they contain a wide variety of antimicrobial factors/activities that can also be harmful to host tissues. A prime example of this dual role is evident in recent work evaluating PMN influx during *S. pneumoniae* infection, where it was shown that early neutrophil influx was essential to controlling infection, but the extended presence of PMNs in the lung led to poor outcome resulting from significant pulmonary damage (Bou Ghanem et al., 2015). Thus, the timing and regulation of neutrophil chemotaxis, turnover, and release of antimicrobial mediators are essential for maintaining pulmonary homeostasis. Pathogenic microbes can disrupt this homeostasis by resisting neutrophil-mediated killing, and suppressing or activating a variety of neutrophil-mediated processes. These activities include but are not limited to modulating neutrophil death, altering the level or timing of neutrophil activation and chemotaxis, and inducing potentially harmful neutrophil-mediated processes. The resulting overzealous neutrophil activation leads to severe tissue damage as a result of the untimely or exacerbated release of toxic agents including the very proteinases, cationic polypeptides, cytokines, and ROS we highlight as potent antimicrobials (Grommes and Soehnlein, 2011; Mizgerd, 2012). The capacity of neutrophils to cause significant pulmonary damage has been recognized for some time. Neutrophil hyper-responsiveness and the dysregulation of neutrophil apoptosis have been shown to contribute to lung injury and poor outcomes in patients with pneumonia clinically and in various animal models (Bordon et al., 2013). Neutrophil depletion has been shown to be protective in a number of infection models, and severe disease almost always correlates with excessive neutrophil influx in the airways, leading to the belief that neutrophils likely play a significant role in ALI (Lee and Downey, 2001; Martin, 2002; Pechous et al., 2013, 2015; Sahoo et al., 2014). Recent bioinformatics analysis during influenza infection of mice revealed a transcriptional signature associated with lethality, and it was further shown that neutrophils are its primary source (Brandes et al., 2013). In addition, the positive feedback of neutrophils recruiting and activating other neutrophils further contributes to lethality by compromising the airways and facilitating the increased release of potentially harmful mediators of host damage (Ichikawa et al., 2013; Quinton and Mizgerd, 2015). Further, the presence of neutrophils and the associated epithelial damage can contribute to spread of pathogens to other tissues, resulting in systemic disease (Bhowmick et al., 2013). Below, we highlight some of the key molecules and processes responsible for combating invading microbes that have also been shown to contribute to disease pathology in the lung, to the detriment of the host. These mediators, therefore, represent key players whose participation in pulmonary immunity must be balanced and regulated in order to avoid exacerbating disease.

### Serine proteases

The proteases utilized by neutrophils to kill microbes can be particularly damaging to host tissues. Accumulation of activated neutrophils in the airways can result in the aberrant and excessive secretion of active proteases into the milieu, resulting in significant lung matrix destruction through their enzymatic activity and relative target promiscuity (Korkmaz et al., 2010). Neutrophil serine proteases can also act as key regulators of innate immune responses in the lung by modifying cytokines and chemokines, as well as by activating key surface receptors that leads to the induction of additional pro-inflammatory cytokines (Grommes and Soehnlein, 2011). Excessive induction of these processes resulting from the continuous dumping of proteases into the milieu in an already inflammatory environment can further contribute to tissue damage in the lung during pneumonia. Of the granule proteins, neutrophil elastase, in particular, has been shown to be a potent mediator of inflammatory damage. Though cathepsin G and proteinase-3 can also contribute to pathology, it is the presence of elastase that is most associated with lung injury associated with infection (Moraes et al., 2006). Infection of elastase-deficient mice with *Burkholderia thailandensis* and *pseudomallei* showed increased survival that correlated with decreased lung tissue damage (Sahoo et al., 2014). Similarly, inhibiting elastase in a hamster model of ALI prevented lung damage from developing (Kawabata et al., 2000). Elevated levels of elastase in the lavage fluid correlate with severity of lung injury during pneumonia in humans, and there exists evidence of a protective effect of inhibiting elastase (Grommes and Soehnlein, 2011). Active elastase is also capable of degrading E-cadherin during lung inflammation, thus potentially contributing to disruption of cell-cell junctions in the lung (Boxio et al., 2016). It's effects can also be less direct, as elastase is known to stimulate lung epithelium release of pro inflammatory cytokines as well as epithelial apoptosis (Chen et al., 2004; Witherden et al., 2004; Moraes et al., 2006; Suzuki et al., 2009). Both of these functions can ultimately lead to increased epithelial permeability, which contributes to pulmonary damage and allows for accelerated transmigration of neutrophils into the alveolar space (Ginzberg et al., 2001). Along with proteinase-3 and cathepsin G, elastase can degrade surfactin proteins D and A, which can prolong inflammation by inhibiting the clearance of apoptotic neutrophils (Vandivier et al., 2002; Moraes et al., 2006). Taken together, the relative promiscuity of neutrophil serine proteases, as well as their ability to directly and indirectly modulate host inflammatory responses highlight their role as key mediators of both pulmonary defense and tissue destruction during severe pneumonia.

### Antimicrobial peptides

The ability of neutrophil antimicrobial peptides to modulate an anti-microbial pro inflammatory response can also make them highly damaging when released in excess. Human defensins can exacerbate pulmonary damage through both the activation of macrophages and the increase of epithelial permeability (Sakamoto et al., 2005; Soehnlein et al., 2008). High concentrations of defensins are typically found in the lavage fluid of patients with ARDS (Ashitani et al., 2004). Prolonged exposure to defensins has been shown to cause epithelial cytotoxicity, and can thus interfere with barrier function (Moraes et al., 2006). This has been seen in transgenic mice expressing defensins, where mice had ALI due to a disrupted capillary-epithelial barrier (Bdeir et al., 2010). Similarly, azurocidin has been shown to play a role in neutrophil-mediated permeability changes in pulmonary tissue, including during *Streptococcus pyogenes* infection (Soehnlein et al., 2008). The antimicrobial polypeptide LL-37 can have cytotoxic effects on nearby endothelial and epithelial cells, and like defensins is known to be highly represented in the lavage fluid of patients with ARDS (Fahy and Wewers, 2005; Aarbiou et al., 2006; Kai-Larsen and Agerberth, 2008). LL-37 is also able to inhibit neutrophil apoptosis, which can result in increased levels of neutrophils present and therefore increased levels of potentially toxic mediators of tissue damage (Barlow et al., 2006).

### ROS

ROS secreted into the phagolysosome are effective as antimicrobials as they can hit any number of targets. This same attribute, though, can be problematic, as ROS can also be released outside of the cell, where they can contribute to disease pathogenesis by interacting with various host targets (Klebanoff, 2005). In the pulmonary compartment, ROS can nonspecifically interact with a number of host molecules and inactivate iron/sulfer proteins, disrupt lipid membranes, crosslink proteins, and cause DNA modification and strand breaks, impairing basic physiological functions (Hampton et al., 1998). ROS are known to be highly damaging to pulmonary tissue, and have been shown to cause significant pulmonary damage and increased permeability in animal models of ALI (Johnson et al., 1981; Auten et al., 2001, 2002) ROS can disrupt intercellular tight junctions by inducing the phosphorylation of focal adhesion kinase in endothelial cells, resulting in a loss of barrier function and increased permeability (Usatyuk and Natarajan, 2005). Inhibition of NADPH oxidase has been shown to alleviate lung injury in a guinea pig model of sepsis-induced lung injury (Wang et al., 1994). Thus, while effective as components of the microbe-containing phagolysosome, excessive release of ROS by necrotic neutrophils or in an attempt to combat infection can cause a great deal of pulmonary injury.

### NETs

The accumulation and prolonged presence of NETs can also be highly damaging to surrounding tissues (Narasaraju et al., 2011). The protein component of NETs, specifically histones, can induce cytotoxic effects upon interaction with lung epithelial and endothelial cells (Saffarzadeh et al., 2012). Accumulation of neutrophils and NETs is known to exacerbate alveolar damage and vascular leakage in mice challenged with a number of pathogens, including influenza A virus (Narasaraju et al., 2011). It has also been established that NET release is responsible for a great deal of collateral damage during *S. pneumoniae* infection (Moorthy et al., 2016; Porto and Stein, 2016). Moreover, *S. pneumoniae* has developed mechanisms to evade trapping and killing by NETs, such that NET release functions only to damage pulmonary tissue rather than facilitate appreciable bacterial clearance (Beiter et al., 2006; Wartha et al., 2007; Moorthy et al., 2016; Porto and Stein, 2016). The presence of neutrophil serine proteases present in NETs can also be a source of tissue destruction in the lung, as their primary antimicrobial function relies on their containment in the phagolysosome. The presence of neutrophil serine proteases in NETs therefore exposes them to various host targets, the cleavage of which can be detrimental to tissue integrity and function. In summary, excessive NET release can be particularly damaging due to the presence of extracellular neutrophil DNA, but also the presence of a variety of toxic proteins and peptides trapped within them.

### Neutrophil resolution

Equally important to neutrophil mediated immunity is the appropriate resolving of neutrophils in the lung. This requires effective coordination of the termination of excessive neutrophil influx and the clearance of apoptotic neutrophils by efferocytosis. These processes are mediated in part by the biosynthesis of specialized pro-resolving mediators such as resolvins, or the presence of other molecules produced by host tissue including extracellular adenosine that signal the need to terminate and resolve an active inflammatory process (Serhan et al., 2002, 2014; Chiang et al., 2012; Bou Ghanem et al., 2015). Neutrophil turnover, specifically by constitutive apoptotic cell death, is an important part of effective pulmonary immunity. Delayed neutrophil death can exacerbate inflammation by increasing neutrophil numbers and therefore their propensity to cause significant tissue damage via the continuous release of toxic ROS, enzymes, and other antimicrobial factors. Effective control of neutrophil cell death is therefore essential to appropriate immune control and resolution of inflammation. During infection, neutrophil lifespan can be prolonged, either by host stimuli in an attempt to better combat infection (Miles et al., 2009), or by pathogens that target neutrophil apoptosis to delay host inflammatory responses or to establish a replicative niche (Stasulli et al., 2015). Further, impaired phagocytosis resulting from either microbial or environmental factors can result in necrotic cell death and the harmful release of granule contacts (Bordon et al., 2013). Effective clearing of dying neutrophils is also highly important to resolving of inflammatory responses, as it can result in the release of toxic enzymes and antimicrobial peptides into the alveolar space. For example, it is known that *Staphylococcus aureus* alpha toxin may contribute to tissue damage during infection by inhibiting macrophage efferocytosis (Cohen et al., 2016). Thus, the directing of neutrophils toward a more inflammatory necrotic pathway coupled with their continued accumulation due to a lack of proper turnover make reduced neutrophil apoptosis a key component of the dysregulation of host responses that lead to pneumonia.

## Targeting neutrophils and host inflammatory responses therapeutically

Efforts to improve the care of patients with pneumonia need to consider key host response factors, including those biomarkers that might predict a poor outcome (Bordon et al., 2013). Though at first glance it may seem counterproductive, it is becoming clear that suppressing potentially harmful aspects of innate immunity in tandem with delivery of appropriate antimicrobial therapy may be beneficial to treating severe pneumonia with some pathogens. The delivery of immunosuppressive drugs or antibodies is often used to treat highly inflammatory disease states, including sepsis and meningitis. Further, corticosteroid infusion has been shown to increase survival in patients with severe community-acquired pneumonia (Confalonieri et al., 2005). The role of neutrophils in the progression of severe pneumonia make them potential targets of neutralization during pulmonary infection. An ideal therapeutic may be one that targets the destructive potential of neutrophils while maintaining their capacity to kill and eliminate invading microbes (Craig et al., 2009). It has been suggested that targeting neutrophil-mediated ALI concurrently with viral growth may be an effective strategy for combating severe influenza pneumonia (Narasaraju et al., 2011). Targeting specific neutrophil components or processes may also yield some benefit. Blocking NET-associated extracellular histones using anti-histone antibodies has been shown to decrease lung pathology during influenza infection (Narasaraju et al., 2011) There is also preclinical and clinical data suggesting that inhibiting neutrophil serine proteases may attenuate some of the deleterious effects of inflammation in the pulmonary compartment (Korkmaz et al., 2010). The success of targeting neutrophils and neutrophil-mediated processes in enhancing survival is likely going to be highly pathogen specific, as neutrophils are absolutely essential for combating infection in many cases. Rather, those pathogens where neutrophils cause considerable tissue destruction, yet do not necessarily affect microbial burden are likely candidates. One example of this is pneumonic plague, where the massive influx of neutrophils into the lung is a hallmark of severe disease, yet depletion of neutrophils has little to no effect on bacterial burden in the lung (Pechous et al., 2013, 2015).

## Thoughts and considerations

Despite the availability of advanced treatment options, severe pneumonia can still result in mortality in upwards of 50% of patients. The precise mechanisms and factors resulting in poor patient outcome during severe pneumonia are not completely understood. This highlights the need to further understand the factors underlying the dysregulation of host inflammatory responses during pneumonia. This includes the failure to initiate an early response, hyper-stimulation, extension of neutrophil lifespan, induction of aberrant granule release, inhibition of neutrophil clearing by macrophages (efferocytosis), and inhibition of neutrophil-mediated killing of pathogens. Neutrophils can play an important role in inducing severe tissue damage in the lung. Thus, a balance must be maintained by employing adequate neutrophil-mediated immune responses without initiating the tissue destruction that can come from their hyper-activity. Further, the timing of neutrophil influx is crucial, as timed depletion studies have revealed that neutrophils can be protective early post-infection, but detrimental when present during later disease stages (Bou Ghanem et al., 2015). The ubiquity of microorganisms in the environment dictates that effective pulmonary immune responses are occurring all the time. This begs the question, then, what tips the balance in one direction or the other? This balance can be disrupted by pathogenic microbes armed with potent stimulatory molecules or pathogenic effectors that counter host innate immune responses to allow for colonization, access key host nutrients, or induce further dissemination. Whether or not neutrophils are protective or detrimental to the host likely depends largely on the invading pathogen. Pathogens may harbor virulence factors that act directly or indirectly on mediators of innate immunity to induce or suppress inflammatory responses. This can either lead to exacerbation of inflammation early during infection, or suppression of host responses until a specific threshold of either pathogen or pathogen product is reached, resulting in damaging hyper-inflammation. In support of this, it has been shown that the timing of neutrophil chemotaxis can dictate the Further, resistance to macrophage and/or neutrophil mediated killing can be an additional virulence mechanism of highly pathogenic organisms. This leads to the continued proliferation of stimulus in the absence of clearance, resulting in the continued influx of innate immune populations into the airways. The evolutionary advantage of inducing this type of host damage is unclear, but also likely depends on the pathogen. Dissemination within the host via increased access to the bloodstream is one immediately obvious scenario. Another may be spread to additional hosts by inducing coughing and thus expulsion of respiratory droplets. Either way, the host-mediated tissue damage induced by neutrophils during infection is an important consideration for both the study of severe pneumonia and the development of advanced therapeutics. It is clear that the attributes that make neutrophils such potent antimicrobial effectors can also be highly damaging to pulmonary tissue. Thus, future efforts to treat severe and lethal pneumonia may involve a tweaking of host inflammatory responses coupled with more traditional antiviral or antibacterial methods.

## Author contributions

The article was conceived of and written by RP.

## Funding

Publication of this article was funded by NIH/NIGMS award P20-GM103625.

### Conflict of interest statement

The author declares that the research was conducted in the absence of any commercial or financial relationships that could be construed as a potential conflict of interest.

## References

[B1] Aarbiou J. Tjabringa G. S. Verhoosel R. M. Ninaber D. K. White S. R. Peltenburg L. T. C.. (2006). Mechanisms of cell death induced by the neutrophil antimicrobial peptides α-defensins and LL-37. Inflamm. Res. 55, 119–127. 10.1007/s00011-005-0062-9 16673155

[B2] Actor J. K. Hwang S.-A. Olsen M. Zimecki M. Hunter R. L. Kruzel M. L. (2002). Lactoferrin immunomodulation of DTH response in mice. Int. Immunopharmacol. 2, 475–486. 10.1016/S1567-5769(01)00189-8 11962727

[B3] Ashitani J.-I. Mukae H. Arimura Y. Sano A. Tokojima M. Nakazato M. (2004). High concentrations of alpha-defensins in plasma and bronchoalveolar lavage fluid of patients with acute respiratory distress syndrome. Life Sci. 75, 1123–1134. 10.1016/j.lfs.2004.01.028 15207659

[B4] Auten R. L. Mason S. N. Tanaka D. T. Welty-Wolf K. Whorton M. H. (2001). Anti-neutrophil chemokine preserves alveolar development in hyperoxia-exposed newborn rats. Am. J. Physiol. Lung Cell Mol. Physiol. 281, L336–L344. 11435208 10.1152/ajplung.2001.281.2.L336

[B5] Auten R. L. Whorton M. H. Nicholas Mason S. (2002). Blocking neutrophil influx reduces DNA damage in hyperoxia-exposed newborn rat lung. Am. J. Respir. Cell Mol. Biol. 26, 391–397. 10.1165/ajrcmb.26.4.4708 11919074

[B6] Bainton D. F. Farquhar M. G. (1968a). Differences in enzyme content of azurophil and specific granules of polymorphonuclear leukocytes. I. Histochemical staining of bone marrow smears. J. Cell Biol. 39, 286–298. 10.1083/jcb.39.2.286 4878049 PMC2107529

[B7] Bainton D. F. Farquhar M. G. (1968b). Differences in enzyme content of azurophil and specific granules of polymorphonuclear leukocytes. II. Cytochemistry and electron microscopy of bone marrow cells. J. Cell Biol. 39, 299–317. 10.1083/jcb.39.2.299 5692583 PMC2107532

[B8] Barlow P. G. Li Y. Wilkinson T. S. Bowdish D. M. E. Lau Y. E. Cosseau C.. (2006). The human cationic host defense peptide LL-37 mediates contrasting effects on apoptotic pathways in different primary cells of the innate immune system. J. Leukoc. Biol. 80, 509–520. 10.1189/jlb.1005560 16793910 PMC1851551

[B9] Bdeir K. Higazi A. A.-R. Kulikovskaya I. Christofidou-Solomidou M. Vinogradov S. A. Allen T. C.. (2010). Neutrophil alpha-defensins cause lung injury by disrupting the capillary-epithelial barrier. Am. J. Respir. Crit. Care Med. 181, 935–946. 10.1164/rccm.200907-1128OC 20093642 PMC2862305

[B10] Beiter K. Wartha F. Albiger B. Normark S. Zychlinsky A. Henriques-Normark B. (2006). An endonuclease allows *Streptococcus pneumoniae* to escape from neutrophil extracellular traps. Curr. Biol. 16, 401–407. 10.1016/j.cub.2006.01.056 16488875

[B11] Belaaouaj A. Kim K. S. Shapiro S. D. (2000). Degradation of outer membrane protein A in Escherichia coli killing by neutrophil elastase. Science 289, 1185–1188. 10.1126/science.289.5482.1185 10947984

[B12] Belaaouaj A. McCarthy R. Baumann M. Gao Z. Ley T. J. Abraham S. N.. (1998). Mice lacking neutrophil elastase reveal impaired host defense against gram negative bacterial sepsis. Nat. Med. 4, 615–618. 10.1038/nm0598-615 9585238

[B13] Bhowmick R. Tin Maung N. H. Hurley B. P. Ghanem E. B. Gronert K. McCormick B. A.. (2013). Systemic disease during *Streptococcus pneumoniae* acute lung infection requires 12-lipoxygenase-dependent inflammation. J. Immunol. 191, 5115–5123. 10.4049/jimmunol.1300522 24089193 PMC3836588

[B14] Bordon J. Aliberti S. Fernandez-Botran R. Uriarte S. M. Rane M. J. Duvvuri P.. (2013). Understanding the roles of cytokines and neutrophil activity and neutrophil apoptosis in the protective versus deleterious inflammatory response in pneumonia. Int. J. Infect. Dis. 17, e76–e83. 10.1016/j.ijid.2012.06.006 23069683

[B15] Borregaard N. Sørensen O. E. Theilgaard-Mönch K. (2007). Neutrophil granules: a library of innate immunity proteins. Trends Immunol. 28, 340–345. 10.1016/j.it.2007.06.002 17627888

[B16] Bou Ghanem E. N. Clark S. Roggensack S. E. McIver S. R. Alcaide P. Haydon P. G.. (2015). Extracellular adenosine protects against *Streptococcus pneumoniae* lung infection by regulating pulmonary neutrophil recruitment. PLoS Pathog. 11:e1005126. 10.1371/journal.ppat.1005126 26313746 PMC4552087

[B17] Boxio R. Wartelle J. Nawrocki-Raby B. Lagrange B. Malleret L. Hirche T.. (2016). Neutrophil elastase cleaves epithelial cadherin in acutely injured lung epithelium. Respir. Res. 17, 129. 10.1186/s12931-016-0449-x 27751187 PMC5067913

[B18] Brandes M. Klauschen F. Kuchen S. Germain R. N. (2013). A systems analysis identifies a feedforward inflammatory circuit leading to lethal influenza infection. Cell 154, 197–212. 10.1016/j.cell.2013.06.013 23827683 PMC3763506

[B19] Brinkmann V. Reichard U. Goosmann C. Fauler B. Uhlemann Y. Weiss D. S.. (2004). Neutrophil extracellular traps kill bacteria. Science 303, 1532–1535. 10.1126/science.1092385 15001782

[B20] Brinkmann V. Zychlinsky A. (2007). Beneficial suicide: why neutrophils die to make NETs. Nat. Rev. Microbiol. 5, 577–582. 10.1038/nrmicro1710 17632569

[B21] Chen H.-C. Lin H.-C. Liu C.-Y. Wang C.-H. Hwang T. Huang T.-T.. (2004). Neutrophil elastase induces IL-8 synthesis by lung epithelial cells via the mitogen-activated protein kinase pathway. J. Biomed. Sci. 11, 49–58. 10.1007/BF02256548 14730209

[B22] Chiang N. Fredman G. Bäckhed F. Oh S. F. Vickery T. Schmidt B. A.. (2012). Infection regulates pro-resolving mediators that lower antibiotic requirements. Nature 484, 524–528. 10.1038/nature11042 22538616 PMC3340015

[B23] Cohen T. S. Jones-Nelson O. Hotz M. Cheng L. Miller L. S. Suzich J.. (2016). S. aureus blocks efferocytosis of neutrophils by macrophages through the activity of its virulence factor alpha toxin. Sci Rep 6:35466. 10.1038/srep35466 27739519 PMC5064327

[B24] Confalonieri M. Urbino R. Potena A. Piattella M. Parigi P. Puccio G.. (2005). Hydrocortisone infusion for severe community-acquired pneumonia: a preliminary randomized study. Am. J. Respir Crit. Care Med. 171, 242–248. 10.1164/rccm.200406-808OC 15557131

[B25] Craig A. Mai J. Cai S. Jeyaseelan S. (2009). Neutrophil recruitment to the lungs during bacterial pneumonia. Infect. Immun. 77, 568–575. 10.1128/IAI.00832-08 19015252 PMC2632043

[B26] Dinauer M. C. Lekstrom-Himes J. A. Dale D. C. (2000). Inherited neutrophil disorders: molecular basis and new therapies. Hematol. Am. Soc. Hematol. Educ. Progr. 2000, 303–318. 10.1182/asheducation-2000.1.303 11701548

[B27] Doss M. White M. R. Tecle T. Hartshorn K. L. (2010). Human defensins and LL-37 in mucosal immunity. J. Leukoc. Biol. 87, 79–92. 10.1189/jlb.0609382 19808939 PMC7167086

[B28] Fahy R. J. Wewers M. D. (2005). Pulmonary defense and the human cathelicidin hCAP-18/LL-37. Immunol. Res. 31, 75–89. 10.1385/IR:31:2:075 15778507 PMC7102303

[B29] Galli S. J. Borregaard N. Wynn T. A. (2011). Phenotypic and functional plasticity of cells of innate immunity: macrophages, mast cells and neutrophils. Nat. Immunol. 12, 1035–1044. 10.1038/ni.2109 22012443 PMC3412172

[B30] Garvy B. A. Harmsen A. G. (1996). The importance of neutrophils in resistance to pneumococcal pneumonia in adult and neonatal mice. Inflammation 20, 499–512. 10.1007/BF01487042 8894714

[B31] Ginzberg H. H. Cherapanov V. Dong Q. Cantin A. McCulloch C. A. Shannon P. T.. (2001). Neutrophil-mediated epithelial injury during transmigration: role of elastase. Am. J. Physiol. Gastrointest. Liver Physiol. 281, G705–G717. 11518683 10.1152/ajpgi.2001.281.3.G705

[B32] Gomez J. C. Wang Q. Doerschuk C. M. (2012). Neutrophils in Acute Bacterial Pneumonia. New York, NY: Springer.

[B33] Good R. A. Quie P. G. Windhorst D. B. Page A. R. Rodey G. E. White J.. (1968). Fatal (chronic) granulomatous disease of childhood: a hereditary defect of leukocyte function. Semin. Hematol. 5, 215–254. 5662845

[B34] Grommes J. Soehnlein O. (2011). Contribution of neutrophils to acute lung injury. Mol. Med. 17, 1. 10.2119/molmed.2010.00138 21046059 PMC3060975

[B35] Hahn I. Klaus A. Janze A.-K. Steinwede K. Ding N. Bohling J.. (2011). Cathepsin G and neutrophil elastase play critical and nonredundant roles in lung-protective immunity against *Streptococcus pneumoniae* in mice. Infect. Immun. 79, 4893–4901. 10.1128/IAI.05593-11 21911460 PMC3232647

[B36] Hampton M. B. Kettle A. J. Winterbourn C. C. (1998). Inside the neutrophil phagosome: oxidants, myeloperoxidase, and bacterial killing. Blood 92, 3007–3017. 9787133

[B37] Ichikawa A. Kuba K. Morita M. Chida S. Tezuka H. Hara H.. (2013). CXCL10-CXCR3 enhances the development of neutrophil-mediated fulminant lung injury of viral and nonviral origin. Am. J. Respir Crit. Care Med. 187, 65–77. 10.1164/rccm.201203-0508OC 23144331 PMC3927876

[B38] Jeyaseelan S. Chu H. W. Young S. K. Worthen G. S. (2004). Transcriptional profiling of lipopolysaccharide-induced acute lung injury. Infect. Immun. 72, 7247–7256. 10.1128/IAI.72.12.7247-7256.2004 15557650 PMC529166

[B39] Jeyaseelan S. Manzer R. Young S. K. Yamamoto M. Akira S. Mason R. J.. (2005). Induction of CXCL5 during inflammation in the rodent lung involves activation of alveolar epithelium. Am. J. Respir. Cell Mol. Biol. 32, 531–539. 10.1165/rcmb.2005-0063OC 15778492 PMC2715322

[B40] Jeyaseelan S. Young S. K. Yamamoto M. Arndt P. G. Akira S. Kolls J. K. (2006). Toll/IL-1R domain-containing adaptor protein (TIRAP) is a critical mediator of antibacterial defense in the lung against *Klebsiella pneumoniae* but not *Pseudomonas aeruginosa*. J. Immunol. 177, 538–547. 10.4049/jimmunol.177.1.538 16785551

[B41] Johnson K. J. Fantone J. C. Kaplan J. Ward P. A. (1981). *In vivo* damage of rat lungs by oxygen metabolites. J. Clin. Invest. 67, 983–993. 10.1172/JCI110149 6894154 PMC370656

[B42] Kai-Larsen Y. Agerberth B. (2008). The role of the multifunctional peptide LL-37 in host defense. Front. Biosci. 13, 3760–3767. 10.2741/2964 18508470

[B43] Kawabata K. Hagio T. Matsumoto S. Nakao S. Orita S. Aze Y.. (2000). Delayed neutrophil elastase inhibition prevents subsequent progression of acute lung injury induced by endotoxin inhalation in hamsters. Am. J. Respir Crit. Care Med. 161, 2013–2018. 10.1164/ajrccm.161.6.9904047 10852782

[B44] Klebanoff S. J. (2005). Myeloperoxidase: friend and foe. J. Leukoc. Biol. 77, 598–625. 10.1189/jlb.1204697 15689384

[B45] Kolaczkowska E. Kubes P. (2013). Neutrophil recruitment and function in health and inflammation. Nat. Rev. Immunol. 13, 159–175. 10.1038/nri3399 23435331

[B46] Korkmaz B. Horwitz M. S. Jenne D. E. Gauthier F. (2010). Neutrophil elastase, proteinase 3, and cathepsin G as therapeutic targets in human diseases. Pharmacol. Rev. 62, 726–759. 10.1124/pr.110.002733 21079042 PMC2993259

[B47] Kreisel D. Nava R. G. Li W. Zinselmeyer B. H. Wang B. Lai J.. (2010). *In vivo* two-photon imaging reveals monocyte-dependent neutrophil extravasation during pulmonary inflammation. Proc. Natl. Acad. Sci. U.S.A. 107, 18073–18078. 10.1073/pnas.1008737107 20923880 PMC2964224

[B48] Lee W. L. Downey G. P. (2001). Neutrophil activation and acute lung injury. Curr. Opin. Crit. Care 7, 1–7. 10.1097/00075198-200102000-00001 11373504

[B49] Levay P. F. Viljoen M. (1995). Lactoferrin: a general review. Haematologica 80, 252–267. 7672721

[B50] Levine A. P. Segal A. W. (2016). The NADPH oxidase and microbial killing by neutrophils, with a particular emphasis on the proposed antimicrobial role of myeloperoxidase within the phagocytic vacuole. Microbiol. Spectr. 4. 10.1128/microbiolspec.MCHD-0018-2015 27726789

[B51] Liou T. G. Campbell E. J. (1995). Nonisotropic enzyme–inhibitor interactions: a novel nonoxidative mechanism for quantum proteolysis by human neutrophils. Biochemistry 34, 16171–16177. 10.1021/bi00049a032 8519774

[B52] Martin T. R. (2002). Neutrophils and lung injury: getting it right. J. Clin. Invest. 110, 1603–1605. 10.1172/JCI0217302 12464663 PMC151642

[B53] Mei J. Liu Y. Dai N. Favara M. Greene T. Jeyaseelan S.. (2010). CXCL5 regulates chemokine scavenging and pulmonary host defense to bacterial infection. Immunity 33, 106–117. 10.1016/j.immuni.2010.07.009 20643340 PMC3748840

[B54] Meng W. Paunel-Görgülü A. Flohé S. Hoffmann A. Witte I. MacKenzie C.. (2012). Depletion of neutrophil extracellular traps *in vivo* results in hypersusceptibility to polymicrobial sepsis in mice. Crit Care 16, R137. 10.1186/cc11442 22835277 PMC3580722

[B55] Miles K. Clarke D. J. Lu W. Sibinska Z. Beaumont P. E. Davidson D. J.. (2009). Dying and necrotic neutrophils are anti-inflammatory secondary to the release of α-defensins. J. Immunol. 183, 2122–2132. 10.4049/jimmunol.0804187 19596979 PMC2948539

[B56] Mizgerd J. P. (2006). Lung infection–a public health priority. PLoS Med. 3:e76. 10.1371/journal.pmed.0030076 16401173 PMC1326257

[B57] Mizgerd J. P. (2008). Acute lower respiratory tract infection. N. Engl. J. Med. 358, 716–727. 10.1056/NEJMra074111 18272895 PMC2711392

[B58] Mizgerd J. P. (2012). Respiratory infection and the impact of pulmonary immunity on lung health and disease. Am. J. Respir Crit. Care Med. 186, 824–829. 10.1164/rccm.201206-1063PP 22798317 PMC3530220

[B59] Moorthy A. N. Rai P. Jiao H. Wang S. Tan K. B. Qin L.. (2016). Capsules of virulent pneumococcal serotypes enhance formation of neutrophil extracellular traps during *in vivo* pathogenesis of pneumonia. Oncotarget 7, 19327–19340. 10.18632/oncotarget.8451 27034012 PMC4991386

[B60] Moraes T. J. Zurawska J. H. Downey G. P. (2006). Neutrophil granule contents in the pathogenesis of lung injury. Curr. Opin. Hematol. 13, 21–27. 10.1097/01.moh.0000190113.31027.d5 16319683

[B61] Müller I. Munder M. Kropf P. Hänsch G. M. (2009). Polymorphonuclear neutrophils and T lymphocytes: strange bedfellows or brothers in arms? Trends Immunol. 30, 522–530. 10.1016/j.it.2009.07.007 19775938

[B62] Narasaraju T. Yang E. Samy R. P. Ng H. H. Poh W. P. Liew A.-A.. (2011). Excessive neutrophils and neutrophil extracellular traps contribute to acute lung injury of influenza pneumonitis. Am. J. Pathol. 179, 199–210. 10.1016/j.ajpath.2011.03.013 21703402 PMC3123873

[B63] Nathan C. (2006). Neutrophils and immunity: challenges and opportunities. Nat. Publ. Group 6, 173–182. 10.1038/nri1785 16498448

[B64] Nathan C. Ding A. (2010). Nonresolving inflammation. Cell 140, 871–882. 10.1016/j.cell.2010.02.029 20303877

[B65] Osman M. O. Kristensen J. U. Jacobsen N. O. Lausten S. B. Deleuran B. Deleuran M.. (1998). A monoclonal anti-interleukin 8 antibody (WS-4) inhibits cytokine response and acute lung injury in experimental severe acute necrotising pancreatitis in rabbits. Gut 43, 232–239. 10.1136/gut.43.2.232 10189850 PMC1727205

[B66] Papayannopoulos V. Metzler K. D. Hakkim A. Zychlinsky A. (2010). Neutrophil elastase and myeloperoxidase regulate the formation of neutrophil extracellular traps. J. Cell Biol. 191, 677–691. 10.1083/jcb.201006052 20974816 PMC3003309

[B67] Pechous R. D. Broberg C. A. Stasulli N. M. Miller V. L. Goldman W. E. (2015). *In vivo* transcriptional profiling of Yersinia pestis reveals a novel bacterial mediator of pulmonary inflammation. MBio 6, e02302–14. 10.1128/mBio.02302-14 25691593 PMC4337571

[B68] Pechous R. D. Sivaraman V. Price P. A. Stasulli N. M. Goldman W. E. (2013). Early host cell targets of Yersinia pestis during primary pneumonic plague. PLoS Pathog. 9:e1003679. 10.1371/journal.ppat.1003679 24098126 PMC3789773

[B69] Pham C. T. N. (2006). Neutrophil serine proteases: specific regulators of inflammation. Nat. Publ. Group 6, 541–550. 10.1038/nri1841 16799473

[B70] Porto B. N. Stein R. T. (2016). Neutrophil extracellular traps in pulmonary diseases: too much of a good thing? Front. Immunol. 7:311. 10.3389/fimmu.2016.00311 27574522 PMC4983612

[B71] Quinton L. J. Mizgerd J. P. (2015). Dynamics of lung defense in pneumonia: resistance, resilience, and remodeling. Annu. Rev. Physiol. 77, 407–430. 10.1146/annurev-physiol-021014-071937 25148693 PMC4366440

[B72] Reeves E. P. Lu H. Jacobs H. L. Messina C. G. M. Bolsover S. Gabella G.. (2002). Killing activity of neutrophils is mediated through activation of proteases by K^+^ flux. Nature 416, 291–297. 10.1038/416291a 11907569

[B73] Rendon A. Rendon-Ramirez E. J. Rosas-Taraco A. G. (2016). Relevant cytokines in the management of community-acquired pneumonia. Curr. Infect. Dis. Rep. 18, 10. 10.1007/s11908-016-0516-y 26874956 PMC7088528

[B74] Restrepo M. I. Anzueto A. (2009). Severe community-acquired pneumonia. Infect. Dis. Clin. North Am. 23, 503–520. 10.1016/j.idc.2009.04.003 19665080

[B75] Saffarzadeh M. Juenemann C. Queisser M. A. Lochnit G. Barreto G. Galuska S. P.. (2012). Neutrophil extracellular traps directly induce epithelial and endothelial cell death: a predominant role of histones. PLoS ONE 7:e32366. 10.1371/journal.pone.0032366 22389696 PMC3289648

[B76] Sahoo M. Del Barrio L. Miller M. A. Re F. (2014). Neutrophil elastase causes tissue damage that decreases host tolerance to lung infection with burkholderia species. PLoS Pathog. 10:e1004327. 10.1371/journal.ppat.1004327 25166912 PMC4148436

[B77] Sakamoto N. Mukae H. Fujii T. Ishii H. Yoshioka S. Kakugawa T.. (2005). Differential effects of alpha- and beta-defensin on cytokine production by cultured human bronchial epithelial cells. Am. J. Physiol. Lung Cell Mol. Physiol. 288, L508–L513. 10.1152/ajplung.00076.2004 15557089

[B78] Schreiber A. Busjahn A. Luft F. C. Kettritz R. (2003). Membrane expression of proteinase 3 is genetically determined. J. Am. Soc. Nephrol. 14, 68–75. 10.1097/01.ASN.0000040751.83734.D1 12506139

[B79] Serhan C. N. Chiang N. Dalli J. Levy B. D. (2014). Lipid mediators in the resolution of inflammation. Cold Spring Harb. Perspect. Biol. 7:a016311. 10.1101/cshperspect.a016311 25359497 PMC4315926

[B80] Serhan C. N. Hong S. Gronert K. Colgan S. P. Devchand P. R. Mirick G.. (2002). Resolvins: a family of bioactive products of omega-3 fatty acid transformation circuits initiated by aspirin treatment that counter proinflammation signals. J. Exp. Med. 196, 1025–1037. 10.1084/jem.20020760 12391014 PMC2194036

[B81] Smart S. J. Casale T. B. (1994a). Pulmonary epithelial cells facilitate TNF-alpha-induced neutrophil chemotaxis. A role for cytokine networking. J. Immunol. 152, 4087–4094. 8144974

[B82] Smart S. J. Casale T. B. (1994b). TNF-alpha-induced transendothelial neutrophil migration is IL-8 dependent. Am. J. Physiol. 266, L238–L245. 8166294 10.1152/ajplung.1994.266.3.L238

[B83] Soehnlein O. Oehmcke S. Ma X. Rothfuchs A. G. Frithiof R. Van Rooijen N.. (2008). Neutrophil degranulation mediates severe lung damage triggered by streptococcal M1 protein. Eur. Respir. J. 32, 405–412. 10.1183/09031936.00173207 18321926

[B84] Standish A. J. Weiser J. N. (2009). Human neutrophils kill *Streptococcus pneumoniae* via serine proteases. J. Immunol. 183, 2602–2609. 10.4049/jimmunol.0900688 19620298

[B85] Stasulli N. M. Eichelberger K. R. Price P. A. Pechous R. D. Montgomery S. A. Parker J. S.. (2015). Spatially distinct neutrophil responses within the inflammatory lesions of pneumonic plague. MBio. 6:e01530-15. 10.1128/mBio.01530-15 26463167 PMC4620470

[B86] Suzuki T. Yamashita C. Zemans R. L. Briones N. Van Linden A. Downey G. P. (2009). Leukocyte elastase induces lung epithelial apoptosis via a PAR-1-, NF-κB-, and p53-dependent pathway. Am. J. Respir. Cell Mol. Biol. 41, 742–755. 10.1165/rcmb.2008-0157OC 19307610 PMC2784410

[B87] Tateda K. Moore T. A. Deng J. C. Newstead M. W. Zeng X. Matsukawa A.. (2001). Early recruitment of neutrophils determines subsequent T1/T2 host responses in a murine model of *Legionella pneumophila* pneumonia. J. Immunol. 166, 3355–3361. 10.4049/jimmunol.166.5.3355 11207291

[B88] Tkalcevic J. Novelli M. Phylactides M. Iredale J. P. Segal A. W. Roes J. (2000). Impaired immunity and enhanced resistance to endotoxin in the absence of neutrophil elastase and cathepsin G. Immunity 12, 201–210. 10.1016/S1074-7613(00)80173-9 10714686

[B89] Usatyuk P. V. Natarajan V. (2005). Regulation of reactive oxygen species-induced endothelial cell-cell and cell-matrix contacts by focal adhesion kinase and adherens junction proteins. Am. J. Physiol. Lung Cell Mol. Physiol. 289, L999–L1010. 10.1152/ajplung.00211.2005 16040628

[B90] Vandivier R. W. Fadok V. A. Hoffmann P. R. Bratton D. L. Penvari C. Brown K. K.. (2002). Elastase-mediated phosphatidylserine receptor cleavage impairs apoptotic cell clearance in cystic fibrosis and bronchiectasis. J. Clin. Invest. 109, 661–670. 10.1172/JCI0213572 11877474 PMC150889

[B91] Wang W. Suzuki Y. Tanigaki T. Rank D. R. Raffin T. A. (1994). Effect of the NADPH oxidase inhibitor apocynin on septic lung injury in guinea pigs. Am. J. Respir. Crit. Care Med. 150, 1449–1452. 10.1164/ajrccm.150.5.7952574 7952574

[B92] Wartha F. Beiter K. Albiger B. Fernebro J. Zychlinsky A. Normark S.. (2007). Capsule and D-alanylated lipoteichoic acids protect *Streptococcus pneumoniae* against neutrophil extracellular traps. Cell Microbiol. 9, 1162–1171. 10.1111/j.1462-5822.2006.00857.x 17217430

[B93] Weinrauch Y. Drujan D. Shapiro S. D. Weiss J. Zychlinsky A. (2002). Neutrophil elastase targets virulence factors of enterobacteria. Nature 417, 91–94. 10.1038/417091a 12018205

[B94] Witherden I. R. Vanden Bon E. J. Goldstraw P. Ratcliffe C. Pastorino U. Tetley T. D. (2004). Primary human alveolar type II epithelial cell chemokine release: effects of cigarette smoke and neutrophil elastase. Am. J. Respir. Cell Mol. Biol. 30, 500–509. 10.1165/rcmb.4890 15033639

[B95] Yamada K. Yanagihara K. Araki N. Harada Y. Morinaga Y. Izumikawa K.. (2011). *In vivo* efficacy of KRP-109, a novel elastase inhibitor, in a murine model of severe pneumococcal pneumonia. Pulm. Pharmacol. Ther. 24, 660–665. 10.1016/j.pupt.2011.08.001 21864700

